# Obinutuzumab Versus Rituximab for the Treatment of Primary Membranous Nephropathy: A Systematic Review and Meta-Analysis

**DOI:** 10.3390/life16030434

**Published:** 2026-03-08

**Authors:** Andrew Lurie, Padideh Daneii, Sana Khan, Zoya Khan, Heena Mansuri, Anas Bizanti

**Affiliations:** Lakeland Regional Health Medical Center, 1324 Lakeland Hills Blvd, Lakeland, FL 33805, USA; padideh.daneii@mylrh.org (P.D.); sana.khan@mylrh.org (S.K.); zoya.khan@mylrh.org (Z.K.); heena.mansuri@mylrh.org (H.M.); anas.bizanti@mylrh.org (A.B.)

**Keywords:** primary membranous nephropathy, Rituximab, Obinutuzumab

## Abstract

**Background:** The benefit of specific B cell-targeted therapy in primary membranous nephropathy has been consistently demonstrated and is part of guideline-directed therapy. Though effective, Rituximab displays a highly variable response rate possibly owing to incomplete peripheral B cell depletion. Obinutuzumab is a second-generation anti-CD20 antibody which offers greater sustained peripheral B cell depletion and thus may result in improved clinical effect. This meta-analysis compares clinical efficacy of Obinutuzumab with Rituximab for the treatment of primary membranous nephropathy. **Methods:** A comprehensive search of PubMed, EMBASE, and Google Scholar was conducted on 10 October 2025. The search identified all studies which directly compared results of Obinutuzumab with Rituximab in the treatment of primary membranous nephropathy. Risk of bias was assessed using the Cochrane ROBINS-I tool. Data extraction and statistical analysis were performed using RevMan 5.1 software, assessing heterogeneity with the I^2^ statistic. **Results:** Ultimately, three retrospective studies including a total of 161 participants were analyzed. The pooled estimated odds ratio for total clinical remission at 6 months was 2.84 (95% CI [1.42–5.69], *p* = 0.003, I^2^ = 0%), and at 12 months was 12.25 ([95% CI 2.67–56.28]), *p* = 0.001, I^2^ = 0%). The pooled estimated odds ratio for complete clinical remission at 6 months was 1.78 (95% CI [0.25–12.63], *p* = 0.57, I^2^ = 0%) at 12 months was 4.12 (95% CI [1.36–12.48], *p* = 0.01, I^2^ = 0%). The pooled estimated odds ratio for immunologic remission at 6 months was 6.18 (95% CI [1.57–24.39], *p* = 0.009, I^2^ = 58%), and at 12 months was 5.56 (95% CI [1.50–20.64], *p* = 0.01, I^2^ = 0%). The pooled estimated odds ratio for peripheral B cell depletion at 6 months was 3.91 (95% CI [0.99–15.40], *p* = 0.05, I^2^ = 25%). **Discussion:** Obinutuzumab signals improvement in clinically relevant end points when compared with Rituximab for the treatment of primary membranous nephropathy but will require confirmation with head-to-head prospective data. The main limitations of this study include small sample sizes, geographic restriction, and retrospective design of the studies resulting in reduced generalizability. **Other:** There was no funding for this study. This review has been registered with PROSPERO (ID 1218735).

## 1. Introduction

Membranous nephropathy (MN) is one of the most common causes of nephrotic syndrome [1]. Primary membranous nephropathy (PMN) occurs when autoantibodies bind to antigens routinely expressed on the glomerular podocyte resulting in glomerular damage and clinical disease [2]. About eighty percent of cases are primary, of which the podocyte autoantigen targeted is the phospholipase-A2 receptor (PLA2R) in 70% of these [3]. Additional minor antigens identified in PMN include thrombospondin type 1 domain-containing 7A (THSD7A), neural epidermal growth factor-like 1 (NELL-1), protocadherin 7 (PCDH7), and neural cell adhesion molecule-1 (NCAM-1) [4]. Identification of the associated autoantigen is a crucial step in clinical management [5]. In anti-PLA2R-related PMN titers correlate with disease activity, and clinical remission is often preceded by immunologic remission; thus, longitudinal monitoring is used to evaluate treatment response [6,7].

PMN has a heterogeneous natural history. If left untreated, roughly 30% of cases result in spontaneous remission, 30% of cases result in nephrotic syndrome with otherwise preserved renal function, and the rest ultimately progress to end-stage renal disease [8]. Treatment guidelines therefore recommend supportive care for all patients and immunosuppression for those who are at elevated risk for progression to loss of kidney function [9]. First-line immunosuppressant regimens include Calcineurin inhibitor +/− glucocorticoids, anti-CD20 therapy with Rituximab, or cyclophosphamide + glucocorticoids (modified Ponticelli regimen).

Rituximab is a type 1 chimeric murine/human type 1 monoclonal antibody targeting CD20 ultimately resulting in lymphocyte cytotoxicity [10]. There are multiple accepted dosing regimens including one dose of 375 mg/m^2^, once-weekly for 375 mg/m^2^ four weeks, and 1–2 g spaced two weeks apart, with additional doses given after six months based on further clinical factors [11,12]. It has proven efficacy to induce remission both as a first-line agent or for refractory disease [13]; however, reports show widely variable response rates [12]. Most reports show nonresponse in up to 20–40% of patients [14]. There is no definitive explanation for the wide range of effect; however, it may relate to inadequate peripheral B cell depletion as similar findings have been shown in related disease states [15]. Moreover, it remains inconclusive whether peripheral B cell depletion adequately reflects presence of B cells at the tissue level [16,17].

Obinutuzumab is a type II fully humanized monoclonal antibody targeting CD20. It has a similar use case and demonstrates great efficacy in the management of Lupus Nephritis [18], whereas Rituximab has historically shown mixed results [19] which may result from superior B cell depleting activity when compared to Rituximab [20,21]. Numerous prior case reports and retrospective studies have documented efficacy of Obinutuzumab in treatment of PMN as both primary treatment and rescue therapy after inadequate response to Rituximab [22]. Unfortunately, these trials have low patient enrollments which reduce the generalizability of the results. As such, though individual cohorts signal clinically meaningful improvements, the data lacks the power needed to guide clinical decision-making. We are therefore conducting a meta-analysis of trials comparing Obinutuzumab to Rituximab [23,24,25] in treatment of primary membranous nephropathy to address this knowledge gap and identify superiority on clinical remission.

## 2. Materials and Methods

### 2.1. Eligibility Criteria

We included all studies evaluating the effect of Obinutuzumab versus Rituximab on the treatment of primary membranous nephropathy. We excluded studies that lacked a control arm or administered Obinutuzumab subsequent to Rituximab (e.g., rescue therapy after failed Rituximab).

### 2.2. Information Sources and Search Strategy

**Figure 1:**
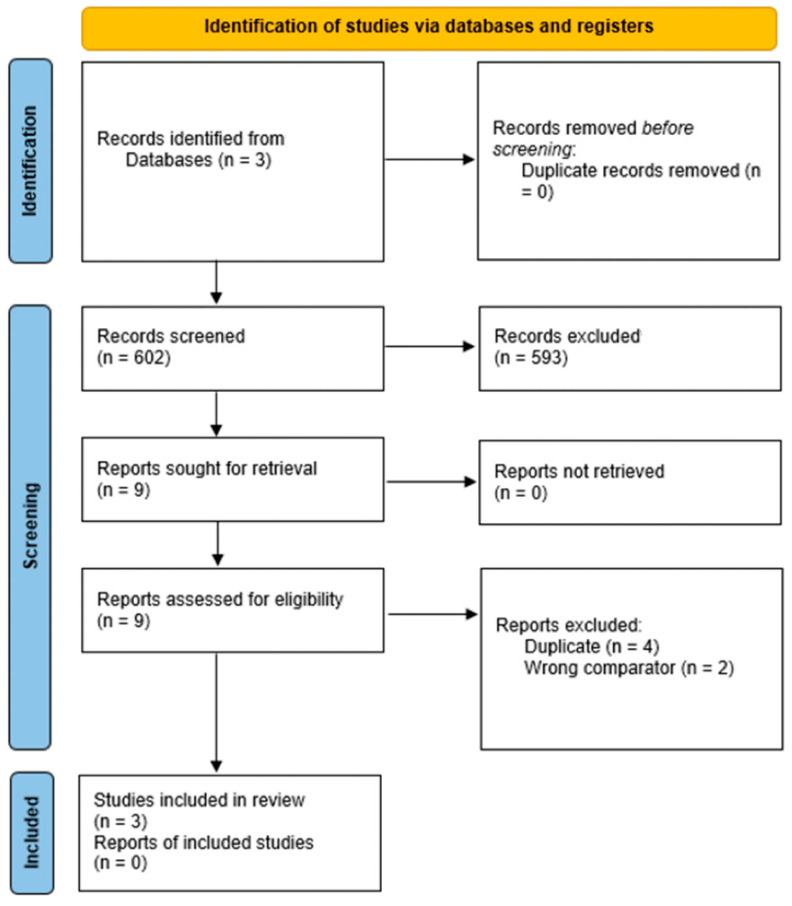
PRISMA flow diagram.

We searched for trials in adherence to the PRISMA guidelines as shown in Figure 1 and the Supplementary Materials. Two investigators (A.L. and S.K.) created a search strategy which was approved by all other investigators. A systematic search of PubMed, Embase-OVID, and Google Scholar was conducted from inception until the date of search on October 10, 2025 without search language restriction. The search terms can be found in detail in the Supplementary Materials.

### 2.3. Selection Process

Two investigators (A.L. and S.K.) independently screened all identified papers for inclusion based on the study title and abstract. After screening, the full text of all studies considered for inclusion were sought and independently assessed by the same investigators. Once all trials had been assessed, any discrepancies, if necessary, were resolved by a third reviewer (A.B.) for final inclusion.

### 2.4. Data Collection Process and Data Extraction

Two investigators (A.L. and P.D.) independently extracted data on the following variables using a standardized data table: site, sample size, design, blinding, randomization, population, comparator, intervention, follow-up duration, primary and secondary endpoints, and inclusion and exclusion criteria. Similarly, clinical characteristics were extracted to a standardized data table including the following: age, sex, peripheral B cell count, hypertension, diabetes, serum albumin, proteinuria, serum creatinine, eGFR, and anti-PLA2R antibody level. We attempted to contact authors for missing data; however, if this was unable to be obtained, there was no replacement or imputation for missing data. Any discrepancies were resolved independently via a third reviewer (A.B.).

### 2.5. Data Items

The primary outcome was clinical remission at 6 months. Clinical remission was defined as (i.) complete remission if urine protein excretion is less than 0.3 g/day with stable kidney function, or (ii.) partial if urine protein excretion is less than 3.5 g/day and reduced by at least 50% from baseline with stable kidney function, and (iii.) combined to create total clinical remission as a composite of complete plus partial remission. Two of the three studies included normalization of serum albumin as requirements for partial remission while one study did not. Secondary outcomes were clinical remission at 12 months, immunologic remission at 6 and 12 months as defined by a reduction in serum anti-PLA2R antibody titers to less than 2 RU/mL, and B cell depletion at 6 months as evaluated by flow cytometry. All results compatible with each outcome domain in each study were sought.

### 2.6. Risk of Bias Assessment

Three investigators (A.L., S.K., and P.D.) independently assessed the risk of bias (RoB) for each trial using the Cochrane ROBINS-I tool [26]. Disagreements were resolved through discussion with a third investigator (A.B.).

### 2.7. Statistical Analysis

We conducted our systematic review based on the 2020 PRISMA guidelines [27] and checklist as shown in the Supplementary Materials. Meta-analysis was performed using the RevMan 5.1 software [28] assessing via the odds ratio. Heterogeneity was assessed via the I^2^ statistic with a value of 50% indicating moderate heterogeneity and 75% significant heterogeneity. We did not perform a sensitivity analysis due to the low number of studies. Certainty of evidence was assessed via the GRADE methodology [29].

## 3. Results

### 3.1. Study Selection

The search strategy has been visualized in Figure 1. A comprehensive search was conducted on October 10 2025 using PubMed, EMBASE-OVID, and Google Scholar to identify relevant citations. The search included the MeSH terms membranous glomerulonephritis, Obinutuzumab, and Rituximab. A similar search was undertaken on EMBASE and Google Scholar databases. The complete search strategy is available in the supplemental file. A total of 602 citations were identified (17 from PubMed, 73 from EMBASE, and 512 from Google Scholar). A total of 593 studies were excluded based on criteria such as not using the intervention or having different controls or population (e.g., previously failed Rituximab). The full text of nine studies was accessed for further review of which 6 studies were excluded due to having the incorrect comparator or duplicate texts. Three (*n* = 3) studies were ultimately selected for the analysis. All three studies were of a retrospective design. Additional information regarding study design is detailed in Table 1, and the baseline characteristics of each individual study are summarized in Table 2.

### 3.2. Study Characteristics

The characteristics of the included studies [23,24,25] are summarized in Table 1. All three trials were conducted in China with inclusion periods spanning from 2015 to 2024. The number of patients in each study was homogenous with small sizes in both groups. The reported mean estimated glomerular filtration rate was similar between groups ranging from 84 to 103. The reported mean 24 h proteinuria varied and ranged from 5.3 to 8.9 g. Follow-up periods varied from six months to two years.

### 3.3. Baseline Characteristics

The baseline characteristics are summarized in Table 2. The median age ranged from 51 to 63 years. Female representation was relatively even, with women comprising between 25 and 50% of the study populations. Hypertension was prevalent with a range of about 40 to 60% of patients, while diabetes was rarer with a range of 15 to 28%. Mean serum albumin ranged from 2.0 to 2.7 g/dL. The mean serum anti-PLA2R antibody titer ranged from 61 to 138 RU/mL. Overall, the populations presented in Table 2 are well-balanced.

### 3.4. Risk of Bias

The three included trials showed some concern for bias primarily due to retrospective design which allowed for inherent bias. Each trial had a small sample size and had a small quantity of missing data from patients lost to follow-up. We did not perform Egger’s test to assess publication bias given that there are only three studies. This risk of bias analysis is summarized in the Supplementary Materials.

### 3.5. Certainty of Evidence

The certainty of evidence table was assessed using the GRADE framework as shown in Table S2. The primary outcome results were considered low quality. The secondary outcome data were considered low quality due to inherent study limitations and indirectness of evidence in part due to retrospective design and inconsistent follow-up duration.

### 3.6. Outcomes

**Figure 2:**
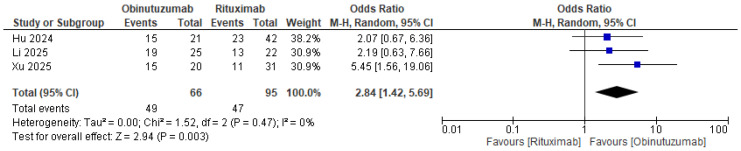
Primary outcome: total clinical remission at 6 months [23,24,25].

The forest plots in Figure 2 and Figures S2–S7 and Table 3 offer a detailed comparison of the effects of Obinutuzumab versus Rituximab in the treatment of primary membranous nephropathy. There was a statistically significant increased odds of total remission at 6 months (OR 2.84 [1.42–5.69], *p* = 0.003, I^2^ = 0%) and 12 months (12.25 [2.67–56.28], *p* = 0.001, I^2^ = 0%), complete remission at 12 months (4.12 [1.36–12.48], *p* = 0.01, I^2^ = 0%), and immunologic remission at 6 months (6.18 [1.57–24.30], *p* = 0.009, I^2^ = 58%) and 12 months (5.56 [1.50–20.64], *p* = 0.01, I^2^ = 0%). There was no significant difference in the odds of complete remission at 6 months (1.78 [0.25–12.63], *p* = 0.57, I^2^ = 0%) or peripheral B cell depletion at 6 months (3.91 [0.99–15.40], *p* = 0.05, I^2^ = 25%).

**Table 3:** Primary and secondary outcomes.

Study Name	Group	Total Clinical Remission 6 Months—No (%)	Total Clinical Remission 12 Months—No (%)	Partial Clinical Remission 6 Months—No (%)	Partial Clinical Remission 12 Months—No (%)	Immunologic Remission 6 Months—No (%)	Immunologic Remission 12 Months—No (%)	Peripheral B Cell Depletion
Hu 2024 [23]	Rituximab	23 (55)	28 (67)	21 (50)	22 (52)	27 (64)	30 (71)	10 (32)
	Obinutuzumab	15 (71)	20 (95)	14 (67)	12 (57)	19 (90)	19 (90)	10 (77)
Xu 2025 [25]	Rituximab	11 (35)	10 (37)	11 (35)	9 (33)	6 (21)	8 (33)	3 (18)
	Obinutuzumab	15 (75)	9 (90)	14 (70)	7 (70)	14 (88)	6 (86)	13 (81)
Li 2025 [24]	Rituximab	13 (59)	-	-	-	14 (64)	-	-
	Obinutuzumab	19 (76)	-	-	-	20 (80)	-	-

## 4. Discussion

This meta-analysis combined data from three retrospective trials evaluating the efficacy of Obinutuzumab versus Rituximab in the treatment of primary membranous nephropathy. The key finding is a higher rate of total clinical remission with Obinutuzumab as compared to Rituximab. This finding signals a clinically significant improvement that could meaningfully affect patient outcomes. Though encouraging, future prospective head-to-head trials will be needed to confirm this hypothesis with greater patient enrollment and higher statistical power. Reassuringly, our findings align with burgeoning changes to clinical practice with increased use of Obinutuzumab. Numerous case reports and retrospective studies have been published suggesting Obinutuzumab as efficacious for both primary treatment and secondary treatment for Rituximab-refractory primary membranous nephropathy [30,31]. This meta-analysis now adds further support for its use.

Contrary to expectations, our findings do not support improvements in peripheral B cell depletion with Obinutuzumab. This result was unusual when taken in context with the prior literature. The landmark LUNAR [19] and MAGESTY [18] trials evaluating Rituximab in Lupus Nephritis suggested suboptimal B cell depletion and early cell line repopulation may underlie the heterogeneity in Rituximab response rates. Thus, the primary motivator for evaluating first versus second-generation anti-CD20 drugs in this context is the hypothesis of a more stable pharmacologic profile resulting in sustained B cell depletion. It is not known why Obinutuzumab would signal greater efficacy without direct evidence of improved B cell depletion. We believe causes of these paradoxical findings may include (i.) inadequate statistical power related to low patient populations, (ii.) inadequate follow-up time to display a true improvement in sustained B cell depletion, or (iii.) inherent inequities between serum B cell depletion and tissue-level B cell depletion. With these several unanswered questions, further direct investigation with Obinutuzumab in primary membranous nephropathy is needed to identify the underlying mechanism.

There are several important limitations of this study. Firstly, all included trials were retrospective, single-center reports conducted in China which introduces selection bias and limits external validity. Similar to the geographic-specific recommendations for mycophenolate and hydroxychloroquine in IgA nephropathy, it cannot be excluded that Obinutuzumab has a different effect size in this population and thus will require global recruitment for further validation. The sample sizes were small, prior immunosuppression regimens were diverse, and follow-up durations were short, preventing the assessment of long-term renal outcomes, relapse rates, or durability of remission. Secondly, there were variable Rituximab dosing regimens which may introduce confounding by indication and effect size inflation. Specifically, differences in Rituximab dosing may reflect disease severity at initial presentation and influence peripheral B cell depletion thus obscuring the evaluation of differential treatment effects between the medications. Thirdly, though similar, there were key differences in definitions of partial remission between the studies. Hu 2024 [23] did not require the normalization of albumin while Xu 2025 [25] and Li 2025 [24] did, thus the true rate of improvement may have been obscured. Fourthly, although the primary endpoint results were statistically significant, the confidence intervals of all three studies were suspiciously wide. Given that odds ratios are known to be unstable in this context, there should be some concern for low statistical rigor which continues to reinforce the need for subsequent prospective data on the subject. More specifically, the results from Xu 2025 [25] have potential to skew data towards a positive result, though with only three total studies completing a sensitivity analysis is unlikely to contribute meaningfully to the dataset due in part to a reciprocal increase in risk of publication bias. Finally, although statistical heterogeneity was low, the small number of studies restricts precision of this calculation and precludes assessment of publication bias.

Future research should focus on prospective, multicenter, randomized controlled trials powered specifically to confirm superiority of Obinutuzumab in primary membranous nephropathy across a diverse patient population. Cost-effectiveness and long-term safety data will also be necessary to guide clinical decision-making.

## 5. Conclusions

Pooled data from three retrospective trials demonstrated positive results with the use of Obinutuzumab in the treatment of primary membranous nephropathy. Obinutuzumab is a promising alternative as either primary treatment or rescue therapy in this population and will require future head-to-head trials to determine its superiority to Rituximab.

## 6. Other Information

This review has been registered with PROSPERO (ID 1218735). A review protocol was not prepared. No amendments were made to the information provided at registration. There was no financial support or sponsors for this study. No author declares any conflicts of interest. All materials prepared for use by the study team may be made available per request.

## Figures and Tables

**Table 1:** Study design.

	Hu 2024 [23]	Li 2025 [24]	Xu 2025 [25]
Country/Sites	China (1 site)	China (1 site)	China (1 site)
Sample Size	63 (21 Obinutuzumab); (42 Rituximab)	47 (25 Obinutuzumab); (42 Rituximab)	51 (20 Obinutuzumab; 31 Rituximab)
Design	Retrospective single center study	Retrospective single center study	Retrospective single center study
Blinding	None	None	None
Randomization	None	None	None
Population	Adults with PMN	Adults with PMN	Adults with PMN refractory to initial treatment
Comparator	Rituximab 375 mg/m^2^ weekly for four doses, and readministered at month 6 if incomplete remission.	Rituximab 1 g every two to three weeks for two total doses	Rituximab 375 mg/m^2^ weekly for one to four doses or 1 g every two weeks for a maximum of two doses
Intervention	Obinutuzumab 1 g every two weeks for two total doses	Obinutuzumab 1 g every two to three weeks for two total doses	Obinutuzumab 1 g every two weeks for a maximum of two doses
Follow-up Duration	12 months	6 months	12 months
Primary Endpoint	Clinical remission	Clinical remission	Clinical remission
Secondary Endpoints	Urine protein excretion, serum albumin, eGFR, peripheral B cell depletion, adverse events	24 h urine protein, serum albumin, immunologic remission, anti PLA2R receptor antibody titer	Complete remission, Immunological remission and safety profile
Key Inclusion Criteria	eGFR 30+ Proteinuria 3.5 g or higher Diagnosis via renal biopsy or serology	eGFR 30+ Diagnosis with serology only	Refractory to initial treatment with glucocorticoid + cyclophosphamide or calcineurin inhibitor OR refractory to initial therapy or with relapse during tapering regimen Diagnosis via renal biopsy or serology
Key Exclusion Criteria	Secondary MN Current treatment with cyclosporine or cyclophosphamide	Use of dialysis, negative serum anti-PLA2R titer, use of other immunosuppressants	Secondary MN

**Table 2:** Baseline characteristics.

Study	Hu 2024 [23]		Xu 2025 [25]		Li 2025 [24]	
	Rituximab	Obinutuzumab	Rituximab	Obinutuzumab	Rituximab	Obinutuzumab
Sample Size	42	21	31	20	22	25
Median age, years (IQR)	55 (33, 64)	51 (45, 63)	56.0 (41.0, 65.0)	56.0 (43.5, 58.8)	63 (55, 69)	51 (40.50, 64)
Male sex—no. (%)	22 (52)	10 (48)	25 (80.6)	15 (75.0)	10 (45.45)	20 (80)
Mean peripheral B cell count, cells/μL (IQR)	288 (200–410)	258 (187–300)	237.5 (133.4, 363.9)	292.8 (192.6, 347.4)	340.58 (232.21, 457.46)	312.61 (202.00, 406.12)
Hypertension	24 (57)	12 (57)	13 (41.9)	10 (50.0)	12 (54.55)	15 (60)
Diabetes	11 (26)	4 (19)	6 (19.4)	3 (15.0)	7 (28)	5 (22.73)
Serum albumin, g/dL (IQR)	2.0 (1.6–2.3)	2.2 (1.7–2.4)	2.7 (2.3, 3.1)	2.6 (2.2, 3.2)	2.69 (2.24, 2.97)	2.59 (2.85 2.87)
Proteinuria, g/24 h (IQR)	7.9 (6.2–10.0)	8.4 (5.5–10.7)	5.3 (2.6, 9.7)	6.6 (3.0, 8.9)	8.09 (5.09, 10.08)	8.90 (7.15, 13.80)
Serum creatinine, μmol/L	-	-	88.4 ± 29.4	77.1 ± 23.9	71.50 (67, 86)	77 (60.50, 88.50)
eGFR, mL/min/1.73 m^2^ (SD)	103 (90–118)	93 (86–109)	86.2 ± 27.3	90.1 ± 20.3	95 (88, 108)	84.85 (75.30, 100.20)
Anti-PLA2R antibody, RU/mL (IQR)	82 (32–161)	61 (50–153)	61.3 (16.3, 137.5)	66.4 (21.8, 273.6)	138.39 (44.84, 320.79)	83.40 (55.90, 344.36)

## Data Availability

The original contributions presented in this study are included in the article/Supplementary Materials. Further inquiries can be directed to the corresponding author.

## Supplementary Materials

The following supporting information can be downloaded at: https://www.mdpi.com/article/10.3390/life16030434/s1.
