# Supplementary material for: Obinutuzumab Versus Rituximab for the Treatment of Primary Membranous Nephropathy: A Systematic Review and Meta-Analysis
Source: Life (Basel). 2026 Mar 8;16(3):434. doi: 10.3390/life16030434 (PMC13028213; doi:10.3390/life16030434)
Supplement: Supplementary file 1 [file life-16-00434-s001.zip › life-4149798-supplementary.pdf]

Supplemental File to Obinutuzumab vs. Rituximab for the treatment of Primary Membranous Nephropathy: A systematic review and meta-analysis

Table of Contents

Search strategy …………….. 2
Risk of bias …………………3
Secondary outcomes………... 4-5
GRADE assessment…………. 6
PRISMA checklist……………7-8

Table S1. Search Strategy

keys	Result number	source
"Glomerulonephritis, Membranous"[Mesh] OR<br>"membranous nephropathy"[tiab] OR<br>"idiopathic membranous nephropathy"[tiab] OR<br>"primary membranous glomerulonephritis"[tiab] OR<br>"membranous glomerulopathy"[tiab]<br>)	7,265	Pubmed
"Obinutuzumab"[Supplementary Concept] OR<br>"obinutuzumab"[tiab] OR<br>"GA101"[tiab] OR<br>"anti-CD20 type II"[tiab]	1,225	Pubmed
"Rituximab"[Mesh] OR<br>"rituximab"[tiab] OR<br>"anti-CD20 monoclonal antibody"[tiab]	36,006	Pubmed
"Remission Induction"[Mesh] OR<br>"clinical remission"[tiab] OR<br>"complete remission"[tiab] OR<br>"partial remission"[tiab] OR<br>"disease remission"[tiab] OR<br>"proteinuria reduction"[tiab]	99,611	Pubmed
("Glomerulonephritis, Membranous"[Mesh] OR<br>"membranous nephropathy"[tiab] OR<br>"idiopathic membranous nephropathy"[tiab] OR<br>"primary membranous glomerulonephritis"[tiab] OR<br>"membranous glomerulopathy"[tiab])<br>AND<br>("Obinutuzumab"[Supplementary Concept] OR<br>"obinutuzumab"[tiab] OR<br>"GA101"[tiab] OR<br>"anti-CD20 type II"[tiab])<br>AND<br>("Rituximab"[Mesh] OR<br>"rituximab"[tiab] OR<br>"anti-CD20 monoclonal antibody"[tiab)<br>AND<br>("Remission Induction"[Mesh] OR<br>"clinical remission"[tiab] OR<br>"complete remission"[tiab] OR<br>"partial remission"[tiab] OR<br>"disease remission"[tiab] OR<br>"proteinuria reduction"[tiab])	17	Pubmed
"membranous nephropathy" OR "idiopathic membranous nephropathy" OR "primary membranous nephropathy" OR "membranous glomerulonephritis" OR "idiopathic membranous glomerulonephritis" OR "primary membranous glomerulonephritis"	19,000	Google scholar
obinutuzumab OR GA101 OR Gazyva OR "anti-CD20 monoclonal antibody" OR "second generation anti-CD20"	38,400	Google scholar
rituximab OR MabThera OR RTX OR "anti-CD20 monoclonal antibody" OR "first generation anti-CD20"	456,000	Google scholar
"clinical remission" OR remission OR "complete remission" OR "partial remission" OR "proteinuria reduction" OR "treatment response" OR "therapeutic response" OR "disease remission"	4,300,000	Google scholar
("membranous nephropathy" OR "idiopathic membranous nephropathy" OR "primary membranous nephropathy") AND (obinutuzumab OR GA101 OR Gazyva) AND (rituximab OR MabThera OR RTX) AND ("clinical remission" OR remission OR "proteinuria reduction")	512	Google scholar
('membranous nephropathy'/exp OR 'membranous glomerulonephritis'/exp OR 'membranous nephropathy' OR 'idiopathic membranous nephropathy' OR 'primary membranous nephropathy' OR 'membranous glomerulonephritis' OR 'idiopathic membranous glomerulonephritis' OR 'primary membranous glomerulonephritis')	14,263	Embase
('obinutuzumab'/exp OR obinutuzumab OR GA101 OR Gazyva OR 'anti-CD20 monoclonal antibody' OR 'second generation anti-CD20')	9,217	Embase
('rituximab'/exp OR rituximab OR MabThera OR RTX OR 'anti-CD20 monoclonal antibody' OR 'first generation anti-CD20')	144,955	Embase
('remission'/exp OR 'clinical remission' OR 'complete remission' OR 'partial remission' OR remission OR 'proteinuria reduction' OR 'treatment response' OR 'therapeutic response' OR 'disease remission' OR 'treatment outcome'/exp)	3,487,136	Embase
('membranous nephropathy'/exp OR 'membranous glomerulonephritis'/exp OR 'membranous nephropathy' OR 'primary membranous nephropathy')<br>AND ('obinutuzumab'/exp OR obinutuzumab OR GA101 OR Gazyva)<br>AND ('rituximab'/exp OR rituximab OR MabThera OR RTX)<br>AND ('remission'/exp OR 'clinical remission' OR 'proteinuria reduction' OR 'treatment response')	73	Embase

Risk of Bias

Figure S1. Risk of bias assessment

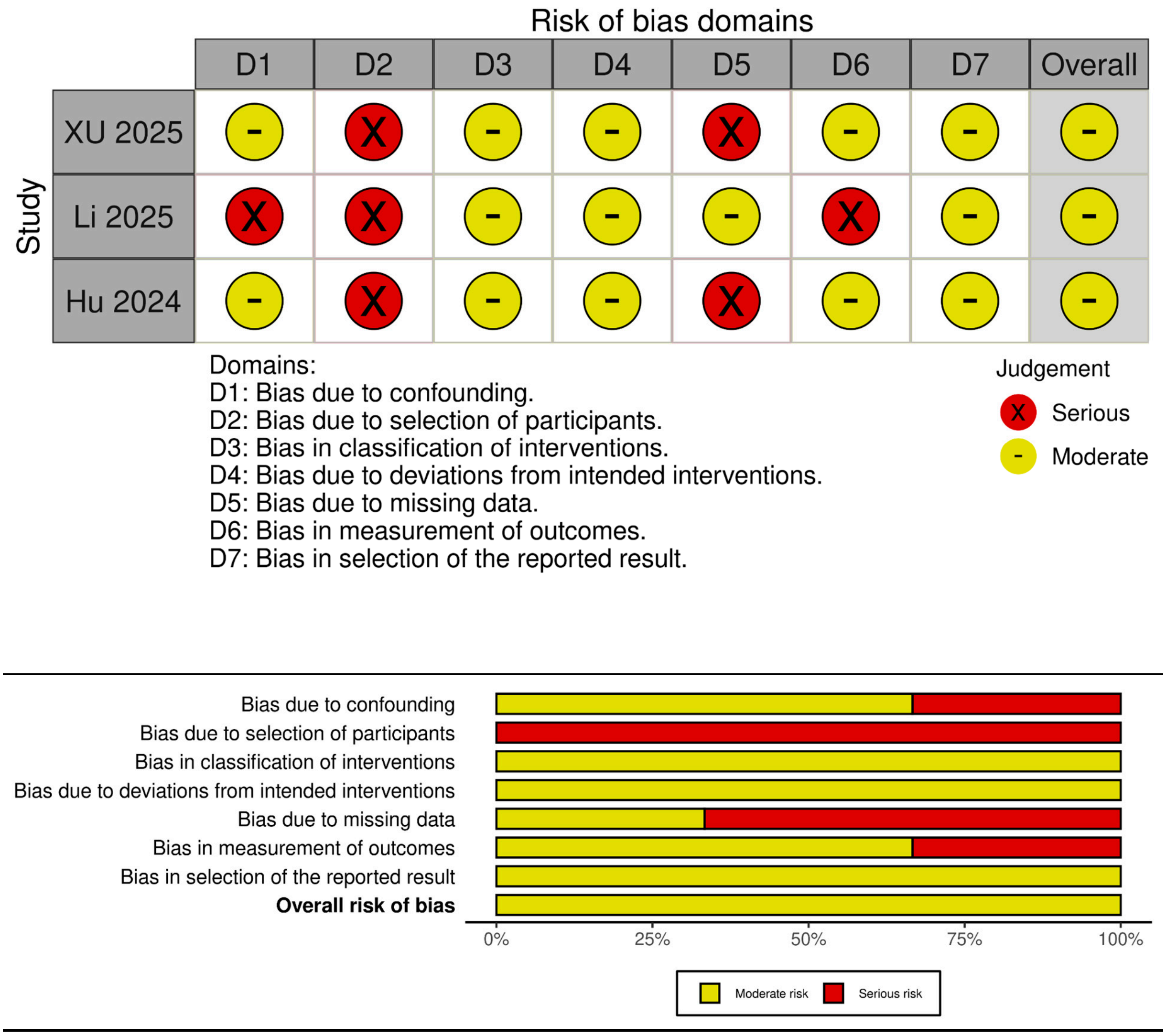

Figure S2. Total clinical remission at 12 months

Study or Subgroup	Obinutuzumab Events	Obinutuzumab Total	Rituximab Events	Rituximab Total	Weight	Odds Ratio M-H, Random, 95% CI
Hu 2024	20	21	28	42	52.3%	10.00 [1.21, 82.35]
Li 2025	0	0	0	0		Not estimable
Xu 2025	9	10	10	27	47.7%	15.30 [1.68, 139.29]
**Total (95% CI)**		**31**		**69**	**100.0%**	**12.25 [2.67, 56.28]**
Total events	29		38			

Heterogeneity: Tau² = 0.00; Chi² = 0.08, df = 1 (P = 0.78); I² = 0%
Test for overall effect: Z = 3.22 (P = 0.001)

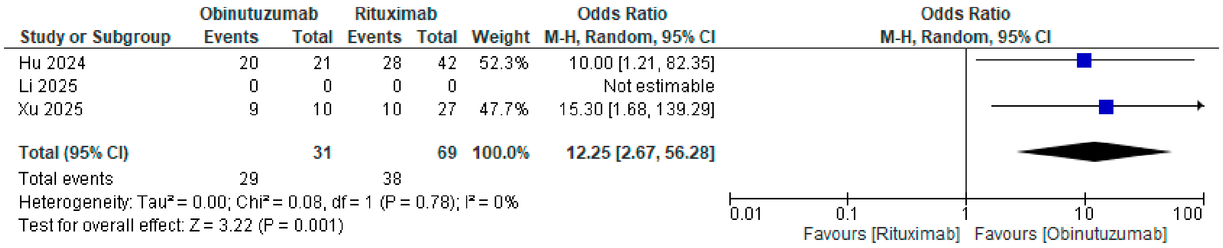

Figure S3. Complete clinical remission at 6 months

Study or Subgroup	Obinutuzumab Events	Obinutuzumab Total	Rituximab Events	Rituximab Total	Weight	Odds Ratio M-H, Random, 95% CI
Hu 2024	1	21	2	42	63.6%	1.00 [0.09, 11.70]
Li 2025	0	0	0	0		Not estimable
Xu 2025	1	20	0	31	36.4%	4.85 [0.19, 124.99]
**Total (95% CI)**		**41**		**73**	**100.0%**	**1.78 [0.25, 12.63]**
Total events	2		2			

Heterogeneity: Tau² = 0.00; Chi² = 0.58, df = 1 (P = 0.45); I² = 0%
Test for overall effect: Z = 0.57 (P = 0.57)

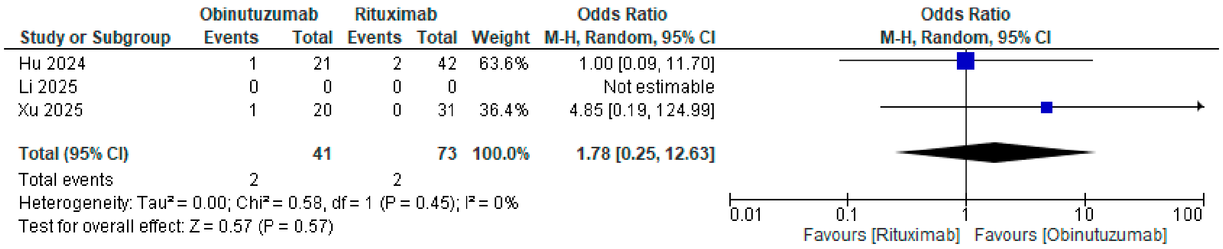

Figure S4. Complete clinical remission at 12 months

Study or Subgroup	Obinutuzumab Events	Obinutuzumab Total	Rituximab Events	Rituximab Total	Weight	Odds Ratio M-H, Random, 95% CI
Hu 2024	8	21	6	42	80.8%	3.69 [1.07, 12.68]
Li 2025	0	0	0	0		Not estimable
Xu 2025	2	10	1	27	19.2%	6.50 [0.52, 81.42]
**Total (95% CI)**		**31**		**69**	**100.0%**	**4.12 [1.36, 12.48]**
Total events	10		7			

Heterogeneity: Tau² = 0.00; Chi² = 0.16, df = 1 (P = 0.69); I² = 0%
Test for overall effect: Z = 2.50 (P = 0.01)

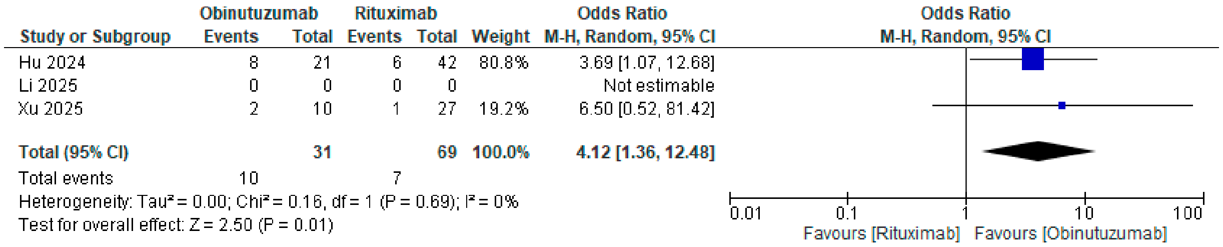

Figure S5. Immunologic Remission at 6 months

Study or Subgroup	Obinutuzumab Events	Obinutuzumab Total	Rituximab Events	Rituximab Total	Weight	Odds Ratio M-H, Random, 95% CI
Hu 2024	19	21	27	42	32.4%	5.28 [1.08, 25.83]
Li 2025	20	25	14	22	37.7%	2.29 [0.62, 8.47]
Xu 2025	14	16	6	28	29.9%	25.67 [4.53, 145.49]
**Total (95% CI)**		**62**		**92**	**100.0%**	**6.18 [1.57, 24.30]**
Total events	53		47			

Heterogeneity: Tau² = 0.85; Chi² = 4.77, df = 2 (P = 0.09); I² = 58%
Test for overall effect: Z = 2.61 (P = 0.009)

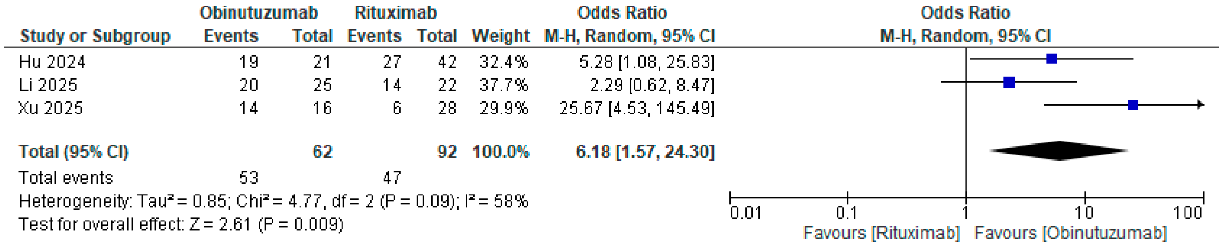

Figure S6. Immunologic Remission at 12 months

Study or Subgroup	Obinutuzumab Events	Obinutuzumab Total	Rituximab Events	Rituximab Total	Weight	Odds Ratio M-H, Random, 95% CI
Hu 2024	19	21	30	42	66.9%	3.80 [0.76, 18.89]
Li 2025	0	0	0	0		Not estimable
Xu 2025	6	7	8	24	33.1%	12.00 [1.23, 117.41]
**Total (95% CI)**		**28**		**66**	**100.0%**	**5.56 [1.50, 20.64]**
Total events	25		38			

Heterogeneity: Tau² = 0.00; Chi² = 0.65, df = 1 (P = 0.42); I² = 0%
Test for overall effect: Z = 2.56 (P = 0.01)

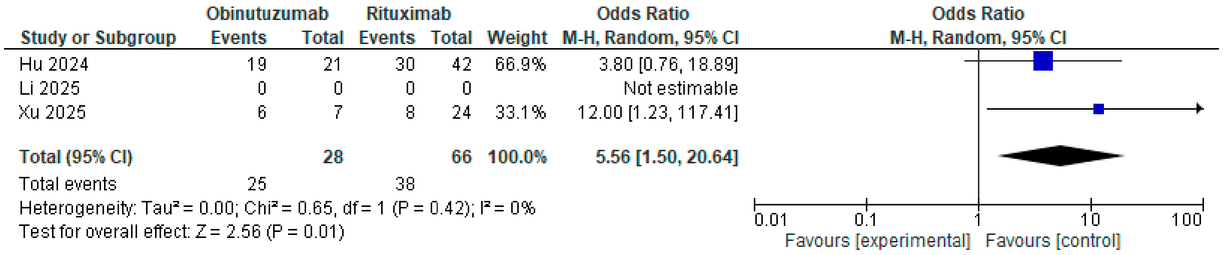

Figure S7. Secondary Outcome: Peripheral B cell depletion at 6 months

Study or Subgroup	Obinutuzumab Events	Obinutuzumab Total	Rituximab Events	Rituximab Total	Weight	Odds Ratio M-H, Random, 95% CI	Year
Hu 2024	10	13	10	31	59.0%	7.00 [1.57, 31.18]	2024
Li 2025	0	0	0	0		Not estimable	2025
Xu 2025	3	7	4	13	41.0%	1.69 [0.25, 11.34]	2025
**Total (95% CI)**		**20**		**44**	**100.0%**	**3.91 [0.99, 15.40]**	
Total events	13		14				

Heterogeneity: Tau² = 0.25; Chi² = 1.33, df = 1 (P = 0.25); I² = 25%
Test for overall effect: Z = 1.95 (P = 0.05)

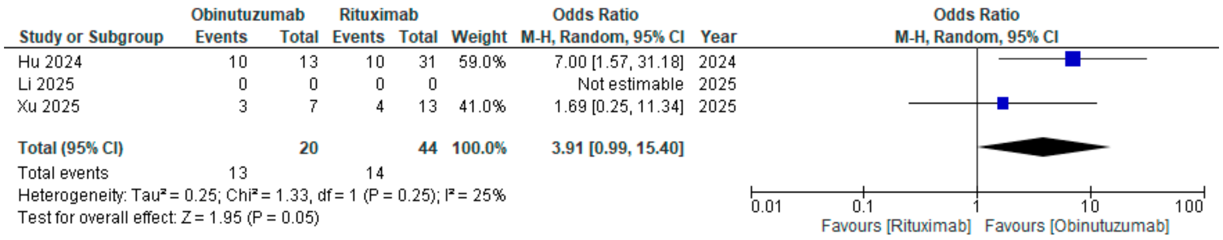

Table S2. GRADE Certainty of Evidence

Outcome	Odds Ratio	Number of Trials	Certainty of Evidence (GRADE)
Total remission (6 months)	2.84 [1.42 - 5.69]	3	⊕⊕⊕○[A, B] MODERATE
Total remission (12 months)	12.25 [2.67 - 56.28]	3	⊕⊕⊕○[A] MODERATE
Peripheral B cell depletion (6 months)	3.91 [0.99 - 15.40]	2	⊕⊕○○ [B] LOW
Immunologic remission (6 months)	6.18 [1.57 - 24.39]	3	⊕⊕⊕○[B] MODERATE
Immunologic remission (12 months)	5.56 [1.50 - 20.64]	2	⊕⊕○○[B] LOW

[A] Inconsistency: similar but slightly different definition of partial remission

[B] Low number of trials: One trial only completed 6 months of follow up

PRISMA

PRISMA 2020 Checklist

Section and Topic	Item #	Checklist item	Location where item is reported
**TITLE**			
Title	1	Identify the report as a systematic review.	1
**ABSTRACT**			
Abstract	2	See the PRISMA 2020 for Abstracts checklist.	1
**INTRODUCTION**			
Rationale	3	Describe the rationale for the review in the context of existing knowledge.	2
Objectives	4	Provide an explicit statement of the objective(s) or question(s) the review addresses.	2
**METHODS**			
Eligibility criteria	5	Specify the inclusion and exclusion criteria for the review and how studies were grouped for the syntheses.	4
Information sources	6	Specify all databases, registers, websites, organisations, reference lists and other sources searched or consulted to identify studies. Specify the date when each source was last searched or consulted.	3
Search strategy	7	Present the full search strategies for all databases, registers and websites, including any filters and limits used.	3
Selection process	8	Specify the methods used to decide whether a study met the inclusion criteria of the review, including how many reviewers screened each record and each report retrieved, whether they worked independently, and if applicable, details of automation tools used in the process.	4
Data collection process	9	Specify the methods used to collect data from reports, including how many reviewers collected data from each report, whether they worked independently, any processes for obtaining or confirming data from study investigators, and if applicable, details of automation tools used in the process.	3-4
Data items	10a	List and define all outcomes for which data were sought. Specify whether all results that were compatible with each outcome domain in each study were sought (e.g. for all measures, time points, analyses), and if not, the methods used to decide which results to collect.	4
	10b	List and define all other variables for which data were sought (e.g. participant and intervention characteristics, funding sources). Describe any assumptions made about any missing or unclear information.	4
Study risk of bias assessment	11	Specify the methods used to assess risk of bias in the included studies, including details of the tool(s) used, how many reviewers assessed each study and whether they worked independently, and if applicable, details of automation tools used in the process.	4
Effect measures	12	Specify for each outcome the effect measure(s) (e.g. risk ratio, mean difference) used in the synthesis or presentation of results.	5
Synthesis methods	13a	Describe the processes used to decide which studies were eligible for each synthesis (e.g. tabulating the study intervention characteristics and comparing against the planned groups for each synthesis (item #5)).	4-5
	13b	Describe any methods required to prepare the data for presentation or synthesis, such as handling of missing summary statistics, or data conversions.	4-5
	13c	Describe any methods used to tabulate or visually display results of individual studies and syntheses.	4-5
	13d	Describe any methods used to synthesize results and provide a rationale for the choice(s). If meta-analysis was performed, describe the model(s), method(s) to identify the presence and extent of statistical heterogeneity, and software package(s) used.	5
	13e	Describe any methods used to explore possible causes of heterogeneity among study results (e.g. subgroup analysis, meta-regression).	n/a
	13f	Describe any sensitivity analyses conducted to assess robustness of the synthesized results.	5
Reporting bias assessment	14	Describe any methods used to assess risk of bias due to missing results in a synthesis (arising from reporting biases).	5
Certainty assessment	15	Describe any methods used to assess certainty (or confidence) in the body of evidence for an outcome.	5
**RESULTS**			
Study selection	16a	Describe the results of the search and selection process, from the number of records identified in the search to the number of studies included in the review, ideally using a flow diagram.	3
	16b	Cite studies that might appear to meet the inclusion criteria, but which were excluded, and explain why they	3

Section and Topic	Item #	Checklist item	Location where item is reported
		were excluded.	
Study characteristics	17	Cite each included study and present its characteristics.	5-7
Risk of bias in studies	18	Present assessments of risk of bias for each included study.	S4
Results of individual studies	19	For all outcomes, present, for each study: (a) summary statistics for each group (where appropriate) and (b) an effect estimate and its precision (e.g. confidence/credible interval), ideally using structured tables or plots.	7-8
Results of syntheses	20a	For each synthesis, briefly summarise the characteristics and risk of bias among contributing studies.	S4
	20b	Present results of all statistical syntheses conducted. If meta-analysis was done, present for each the summary estimate and its precision (e.g. confidence/credible interval) and measures of statistical heterogeneity. If comparing groups, describe the direction of the effect.	7-8
	20c	Present results of all investigations of possible causes of heterogeneity among study results.	7-8
	20d	Present results of all sensitivity analyses conducted to assess the robustness of the synthesized results.	n/a
Reporting biases	21	Present assessments of risk of bias due to missing results (arising from reporting biases) for each synthesis assessed.	n/a
Certainty of evidence	22	Present assessments of certainty (or confidence) in the body of evidence for each outcome assessed.	S4
**DISCUSSION**			
Discussion	23a	Provide a general interpretation of the results in the context of other evidence.	10
	23b	Discuss any limitations of the evidence included in the review.	10
	23c	Discuss any limitations of the review processes used.	10
	23d	Discuss implications of the results for practice, policy, and future research.	10
**OTHER INFORMATION**			
Registration and protocol	24a	Provide registration information for the review, including register name and registration number, or state that the review was not registered.	10
	24b	Indicate where the review protocol can be accessed, or state that a protocol was not prepared.	10
	24c	Describe and explain any amendments to information provided at registration or in the protocol.	10
Support	25	Describe sources of financial or non-financial support for the review, and the role of the funders or sponsors in the review.	10
Competing interests	26	Declare any competing interests of review authors.	10
Availability of data, code and other materials	27	Report which of the following are publicly available and where they can be found: template data collection forms; data extracted from included studies; data used for all analyses; analytic code; any other materials used in the review.	10
